# Relation between insertion torque and tactile, visual, and rescaled gray value measures of bone quality: a cross-sectional clinical study with short implants

**DOI:** 10.1186/s40729-019-0158-6

**Published:** 2019-02-11

**Authors:** Diego Fernandes Triches, Fernando Rizzo Alonso, Luis André Mezzomo, Danilo Renato Schneider, Eduardo Aydos Villarinho, Maria Ivete Rockenbach, Eduardo Rolim Teixeira, Rosemary Sadami Shinkai

**Affiliations:** 1 Postgraduate Program in Dentistry, Pontifical Catholic University of Rio Grande do Sul (PUCRS), Dental School, Avenida Ipiranga, 6681 – Prédio 6, Porto Alegre, RS 90619-900 Brazil; 2 Postgraduate Program in Dentistry, Federal University of Santa Catarina (UFSC), Florianópolis, Brazil

**Keywords:** Bone quality, Insertion torque, Primary stability, Short implants

## Abstract

**Background:**

This study assessed the relationship between insertion torque and bone quality evaluated during surgery and in preoperative computed tomographic (CT) images analyzed either visually or by rescaled mean gray values (MGVs). The study also tested the correlation between the clinical and radiographic measures of bone quality.

**Methods:**

The consecutive sample was composed of 45 short implants (4.1 × 6 mm) placed in the posterior region of 20 patients. Intra-surgical tactile bone quality, based on the classification of bone types by Lekholm and Zarb, and insertion torque were recorded during the implant placement. Visual bone quality and normalized MGV were assessed in standardized axial, coronal, and sagittal sections of preoperative CT images. Data were analyzed by ANOVA and Spearman correlation (alpha = 0.05).

**Results:**

Insertion torque was associated with all assessment methods of bone quality (tactile, CT visual, MGV). A moderate correlation was found among all methods of bone quality, except for CT visual assessment and tactile evaluation. MGVs varied as a function of arch, dental region, insertion torque, and bone types.

**Conclusions:**

The results suggest that bone quality measures affect primary stability as recorded by insertion torque, and the assessment methods are consistently related.

## Background

The early clinical success of short implants can be affected by poor bone quality and low primary stability because implant micromovement can promote the formation of a fibrous capsule during the osseointegration process. It has been reported that the greater the insertion torque, the greater the resistance of the bone-implant interface to the shear forces that tend to rotate the implant [1]. Clinically, insertion torque is the most practical method for measuring primary stability and can be recorded with the manual torque wrench or contra-angle and motor used for implant placement. Resonance frequency and damping capacity have also been used to measure the primary and secondary stability of short implants in research [2], but the procedures are more complex and require sophisticated equipment and extra clinical time.

Bone quality is a generic term for the characterization of bone tissue in three dimensions: the structural quality, related to the amount of the cortical bone and to the trabecular bone pattern; the bone density, related to the amount of bone mineralization and/or the amount of bone by its volume; and the amount of bone, related to the volume of bone available [3]. Bone quality varies intra- and inter-subject depending on the thickness of cortical bone, the amount of trabecular bone, and the amount of bone tissue mineralization in the region of interest for implant placement [4]. Some classifications of bone quality have considered cortical bone thickness and trabecular bone structure based on preoperative panoramic radiographs and tactile perception during exploratory drilling of the implant site; bone density based on the tactile sensation and Hounsfield units of computed tomography (CT) images; radiographic pattern of trabecular bone; and intra-surgical bone density and biopsy with histomorphometric evaluation [5–8]. However, most classifications of bone quality for routine clinical use still are not validated by using objective and subjective assessment methods.

Both multislice CT and cone beam CT are used for presurgical assessment of bone density and quality [4–11]. There is a strong correlation between gray values in cone beam CT and Hounsfield units in multislice CT [4, 12–14]. The visual inspection of CT sections avoids the superimposition of anatomical structures seen in radiographs; thus, the region of interest in trabecular bone can be evaluated without the interference of cortical bone. Positive associations of primary stability with bone density [15], bone volume [16], and thickness of the cortical bone [17] have been reported. If it were possible to accurately relate bone quality measures with primary implant stability, the surgical, prosthetic, and loading planning could be more precise and predictable.

Therefore, this study aimed at assessing the relationship between insertion torque and bone quality evaluated by intra-surgical tactile perception and in preoperative CT images analyzed either visually or by rescaled mean gray values. The association among the subjective and objective measures of bone quality also was tested. The null hypothesis is that there is no relation between insertion torque, visual, and rescaled gray values of the bone in this sample of short implants.

## Methods

This study reports cross-sectional, correlational data of a prospective clinical research project [2] approved by the university Institutional Review Board (10/05074). The research protocol followed the precepts of the Declaration of Helsinki and its amendments. All patients signed an informed consent form.

A consecutive, non-probabilistic sample consisted of 45 implants placed in 20 patients treated by experienced specialists in oral implantology in a private clinic setting. Inclusion criteria were adult patients in need for implant-supported single crowns in the posterior region of the maxilla and mandible and indication of 6-mm long implants, with 2 mm of safety margins for the mandibular canal, lingual cortex of submandibular fossa, and maxillary sinus. Patients were excluded according to the following criteria: previous osseointegration failure or pathologic lesions in the region of interest, use of bone graft or biomaterials, use of bisphosphonates, heavy smoking habit (up to 10 cigarettes/day), non-controlled diabetes, immunosuppression, local radiotherapy, active periodontal disease, poor oral hygiene, or use of removable prosthesis in the antagonist arch.

Clinical data were collected by means of anamnesis, physical examination, and preoperative CT images for surgical planning. Data on implant characteristics and insertion torque were collected at the surgery session.

### Surgical protocol and insertion torque measurement

A total of 45 Standard Plus Regular Neck SLActive® implants (Straumann AG, Basel, Switzerland), 6-mm long and 4.1-mm diameter, were installed in 20 patients. The non-submerged, one-stage surgical protocol was adopted.

Preoperative asepsis of the face and oral cavity was performed with 0.12% chlorhexidine. After local anesthesia with 4% articaine hydrochloride with adrenaline 1:100,000, an incision was made on the ridge crest with total detachment of the flap.

**Fig. 1:**
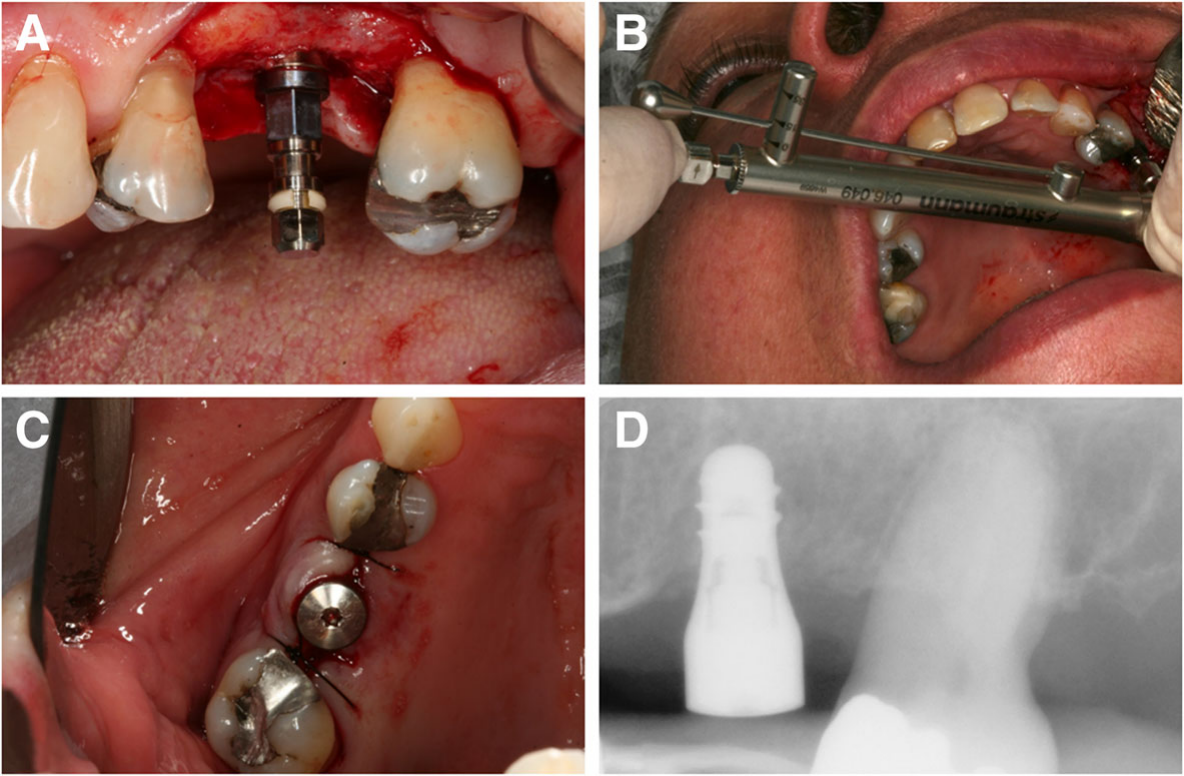
Clinical case of a short implant (4.1 × 6 mm) placed in the region of the left maxillary first molar. **a** Implant installed. **b** Insertion torque measurement using the manual torque wrench. **c** Implant with healing cap and flap suture. **d** Immediate periapical radiograph after surgery

With a 16:1 counter-angle (KaVo Dental®, Biberach, Germany) coupled on an electric motor (Smart® Driller, Jaguaré, São Paulo, Brazil), at a rotation speed of 900 rpm, the surgical milling sequence (1.4-mm spherical drill, 2.3-mm spherical drill, 2.2-mm helical drill, 2.8-mm helical drill, and 3.5-mm helical drill) was performed, with no use of a countersink drill or bone tap. The implant was inserted to the limit between the treated surface of the threads and the smooth platform surface, by using the contra-angle with adapter, at a speed of 18 rpm (Fig. 1a).

The insertion torque was measured using the manual torque wrench (Straumann Dental Implant System®, Waldenburg, Switzerland) (Fig. 1b), according to three categories: < 15 N cm, 15 to 35 N cm, and > 35 N cm. A healing cap was installed, and the suture was made with nylon 5-0 (Fig. 1c). The patients were prescribed with antibiotics (amoxicillin 500 mg, 8/8 h for 7 days), anti-inflammatory drugs (nimesulide 100 mg, 12/12 h for 4 days), and mouthwash with 0.12% chlorhexidine digluconate for 15 days. The sutures were removed after 1 week.

### Intra-surgical tactile evaluation of bone quality (bone types)

During the drilling for implant placement, the surgeon used his tactile perception to assess the bone ridge. The surgeon considered the thickness of the cortical layer and the resistance of the trabecular bone to categorize the bone into four types, based on the classification of Lekholm and Zarb [5]: type 1 (large homogeneous cortical bone and little trabecular bone), type 2 (thick cortical layer surrounding a dense trabecular bone), type 3 (thin cortical layer surrounding a dense trabecular bone), and type 4 (thin cortical layer surrounding a sparse trabecular bone). All surgeries were performed by the same previously trained surgical team.

### Preoperative computed tomography images

Preoperative diagnostic CT images were acquired in the Digital Imaging and Communications in Medicine (DICOM) protocol, and one single cone beam scanner (i-CAT, Imaging Sciences Intl, Hatfield, PA, USA) and one single multislice scanner (Elscint CT Twin II, Elscint Ltd., Haifa, Israel) were used in this study. The DICOM images were reconstructed with the ImageJ software (version 1.51; National Institute of Health, Bethesda, MD, USA) for bone quality evaluation of the regions of interest (ROIs).

**Fig. 2:**
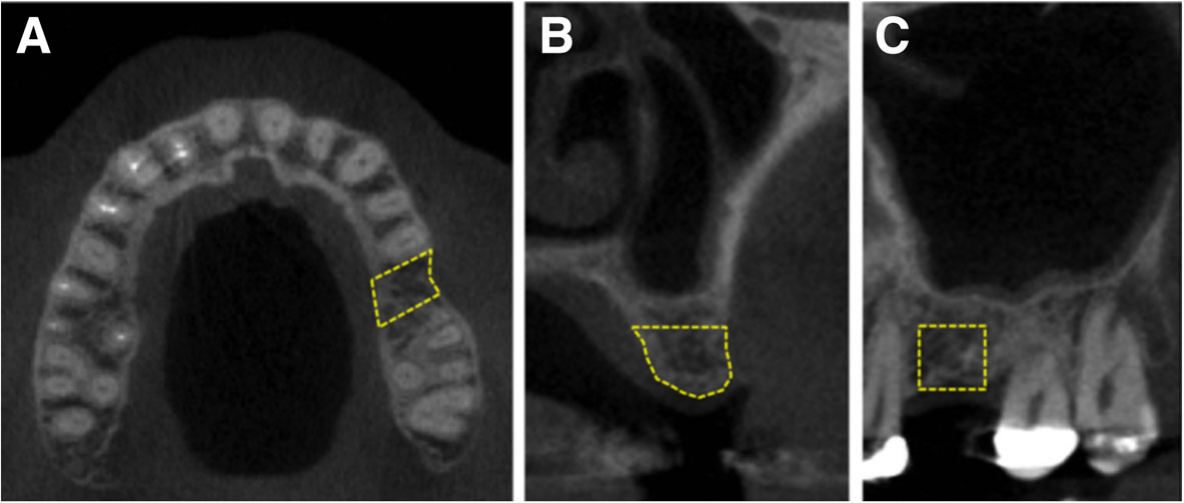
Preoperative CT image showing the site for the definition of the ROI (simulated area delimited by a yellow dashed line for illustration purpose) in the axial (**a**), coronal (**b**), and sagittal (6 × 6 mm) (**c**) sections

A standardized digital periapical radiograph was obtained after suture removal and used to measure the distance from the actual implant center to the proximal side of the nearest tooth at bone level. Using this reference distance, the ROIs in the 1-mm thick CT slices were manually traced corresponding to the alveolar bone (cortical and trabecular bones) in the axial, coronal, and sagittal sections as follows (Fig. 2):

- Axial ROI: Using the reference implant location line, the ROI was defined as the alveolar bone area with a 6-mm width corresponding to 3 mm on each side of the future implant center, including the buccal and lingual cortical layers.
- Coronal ROI: Area defined by the outer border of the cortical bone with a 6-mm height and a line joining the buccal and lingual cortical layers.
- Sagittal ROI: A 6 × 6 mm square was defined as the area corresponding to the implant plus 1 mm of the surrounding bone at the mesial and distal sides.

### Visual assessment of bone quality (bone types)

The visual evaluation of bone quality in the preoperative CT images was performed by a senior, calibrated, and experienced radiologist in CT, who was blind to the other measures of bone quality and insertion torque. After memorizing the ROIs traced in the axial, coronal, and sagittal CT sections, the boundary lines were removed to avoid any interference during the visual assessment. The cortical bone was defined as a white and homogeneous outer layer of the alveolar ridge. The trabecular bone was defined as the structure between the two cortical layers. The examiner analyzed the images as many times as needed to categorize each implant site into bone types 1, 2, 3, or 4, according to the classification of Lekholm and Zarb [5].

### Measurement of mean gray values

The DICOM files were imported to the ImageJ software, and the original axial stacks were used for CT image normalization in 32-bit [18]. Two 5 × 5 mm squares were delimitated for air and soft tissue in the same axial slice containing the ROI (cortical plus trabecular bones).

The original CT scans were rescaled applying the following formula:

$$ {\mathrm{GV}}_n=-{\mathrm{GV}}_{\mathrm{air}}\times \frac{1000}{{\mathrm{GV}}_{\mathrm{st}}-{\mathrm{GV}}_{\mathrm{air}}} $$

where −GV_air_ is the mean gray value for air and GV_st_ is the mean gray value for a central soft tissue square. This calibration was performed by subtracting the gray value for air and multiplying the result by the ratio of 1000 divided by the result of the gray value for soft tissue minus the gray value for air. As a result of this gray value transformation, GV_air_ = 0 and GV_st_ = 1000 for all CT images.

The mean gray value of each rescaled ROI was measured on the three orthogonal planes: axial, coronal, and sagittal, totaling three ROIs per implant site. The average of the rescaled gray values for the three ROIs was computed for each implant site.

### Statistical analysis

Data were analyzed by descriptive and inferential statistics using the software XLSTAT version 2018 (Addinsoft SARL, New York, USA), and a two-tailed significance level of 0.05 was adopted. Spearman correlation coefficients were used to test the association among assessment methods of bone quality (intra-surgical tactile evaluation, CT visual assessment, mean gray values) and primary stability (insertion torque). One-way ANOVA followed by Tukey HSD was used to test the variation of mean gray values (average of ROIs) as a function of arch (maxilla, mandible), dental region (premolar, molar), insertion torque (< 15 N cm, 15 to 35 N cm, > 35 N cm), and bone types (1, 2, 3, 4) as classified by CT visual assessment and by intra-surgical tactile evaluation.

## Results

Descriptive statistics of the sample are shown in Table 1. For statistical analysis, some data were missing: one implant had mobility after surgery and was lost and four CT scans, containing 11 ROIs, were not used for bone quality analysis due to technical problems. As only one case was categorized as bone type 1 by CT visual or by tactile evaluation, the corresponding mean gray values were excluded for the ANOVA tests. Shapiro-Wilk tests showed that the data on mean gray values for all three ROIs and average values followed normal distributions.

**Table 1:** Descriptive statistics of the sample

Variable	Frequency	Mean	SD	95% confidence interval
Patient	20			
Sex (female)	12			
Age (years)		52	12	[46–58]
Implant per arch				
Maxilla	22			
Mandible	22			
Implant per region				
Premolar	10			
Molar	34			
Insertion torque				
< 15 N cm	17			
15 to 35 N cm	15			
> 35 N cm	12			
Tactile evaluation				
Bone type 1	1			
Bone type 2	8			
Bone type 3	21			
Bone type 4	14			
CT visual evaluation				
Bone type 1	1			
Bone type 2	5			
Bone type 3	16			
Bone type 4	11			
CT mean gray values				
ROI axial		1581	241	[1499–1664]
ROI coronal		1560	220	[1485–1636]
ROI sagittal		1373	205	[1303–1443]
Average of ROIs		1505	206	[1434–1576]

Table 2 shows that insertion torque had significant correlation with all assessment methods of bone quality. A moderate association was found among all methods to assess bone quality, except for CT visual and intra-surgical tactile evaluation.

**Table 2:** Matrix of Spearman correlation coefficients and *P* values (in brackets) for the association among assessment methods of bone quality (intra-surgical tactile perception, preoperative CT visual evaluation, preoperative CT mean gray values (MGVs)) and primary stability (insertion torque)

Variables	Torque	Tactile	CT visual	MGV_avg	MGV_axial	MGV_cor	MGV_sag
Torque	–	− 0.770 (< 0.001)	− 0.415 (0.017)	0.677 (< 0.001)	0.620 (< 0.001)	0.629 (< 0.001)	0.607 (< 0.001)
Tactile	− 0.770 (< 0.001)	–	0.342 (0.052)	− 0.670 (< 0.001)	− 0.643 (< 0.001)	− 0.697 (< 0.001)	− 0.469 (0.006)
CT visual	− 0.415 (0.017)	0.342 (0.052)	–	− 0.516 (0.002)	− 0.518 (0.002)	− 0.483 (0.005)	− 0.421 (0.015)
MGV_avg	0.677 (< 0.001)	− 0.670 (< 0.001)	− 0.516 (0.002)	–	0.970 (< 0.001)	0.939 (< 0.001)	0.848 (< 0.001)
MGV_axial	0.620 (< 0.001)	− 0.643 (< 0.001)	− 0.518 (0.002)	0.970 (< 0.001)	–	0.926 (< 0.001)	0.741 (< 0.001)
MGV_coronal	0.629 (< 0.001)	− 0.697 (< 0.001)	− 0.483 (0.005)	0.939 (< 0.001)	0.926 (< 0.001)	–	0.671 (< 0.001)
MGV_sagittal	0.607 (< 0.001)	− 0.469 (0.006)	− 0.421 (0.015)	0.848 (< 0.001)	0.741 (< 0.001)	0.671 (< 0.001)	–

There was a significant difference in rescaled mean gray values (average) as a function of arch (mandible greater than maxilla), dental region (premolar greater than molar), insertion torque (greater in higher torque, > 35 N cm, than in low torques, < 15 N cm), and bone quality (type) as categorized by tactile evaluation (bone types 2 and 3 greater than type 4) and CT visual assessment (bone type 2 greater than types 3 and 4) (Table 3).

**Table 3:** Comparison of CT mean gray values (average of the axial, coronal, and sagittal ROIs) as a function of arch, dental region, insertion torque, and bone types as classified by CT visual assessment and by intra-surgical tactile evaluation

Variable	Mean^a^	Std error	95% confidence interval	*P* value (F; DF)
Arch				< 0.001
Maxilla	1358A	38.1	[1281–1436]	(27.974; 1)
Mandible	1636B	36.1	[1563–1709]	
Dental region				0.014
Premolar	1661A	68.7	[1521–1800]	(6.641; 1)
Molar	1460B	36.7	[1386–1535]	
Insertion torque				0.002
< 15 N cm	1375A	49.5	[1274–1476]	(7.595; 2)
15 to 35 N cm	1510AB	49.5	[1409–1611]	
> 35 N cm	1667B	56.4	[1552–1782]	
Tactile evaluation				0.001
Bone type 2	1650A	60.0	[1528–1772]	(8.703; 2)
Bone type 3	1537A	43.8	[1447–1626]	
Bone type 4	1341B	49.0	[1242–1441]	
CT visual evaluation				0.015
Bone type 2	1742A	83.4	[1571–1912]	(4.828; 2)
Bone type 3	1495B	46.6	[1399–1590]	
Bone type 4	1434B	56.2	[1318–1549]	

^a^Means followed by distinct letters are statistically different at a significance level of 0.05 (one-way ANOVA and pairwise comparisons by Tukey HSD)

## Discussion

This study showed that low bone quality, as assessed by clinical and image methods, is related with low primary stability of 6-mm short implants placed at the posterior region of the maxilla and mandible. Higher insertion torque values were associated with better bone types and higher mean gray values in CT images. Insertion torque had a negative moderate association with bone type categorization by intra-surgical tactile evaluation and visual assessment of CT images. In addition, a positive moderate association was found between insertion torque and average mean gray values, as well as for segmental mean gray values in the axial, coronal, and sagittal ROIs.

The present study assessed the variation in normalized mean gray values to evaluate the bone quality in preoperative CT images using the axial, coronal, and sagittal sections, and the average of the three ROIs. Several studies have investigated the potential clinical application of CBCT mean gray values, especially for bone density evaluation in comparison with various clinical bone parameters [19–27]. However, before using CBCT gray values for bone density estimations, a histogram calibration is needed.

One single cone beam scanner (i-CAT, Imaging Sciences Intl, Hatfield, PA, USA) and one single multislice scanner (Elscint CT Twin II, Elscint Ltd., Haifa, Israel) were used in this study. Even though quantitative differences in absolute numbers might be expected using distinct imaging modalities, a clinical approach was established to clinically comparable values. Using rescaled gray values through a pseudo-Hounsfield scale, the small differences between them were minimized.

In addition to the known CBCT exposure factors that contribute to the deviation of gray values, e.g., noise, beam hardening, limited FOV, local tomography effect, and the position inside the FOV, the machines appear to have incorporated a “histogram shift” in their reconstruction algorithm. This implies that the gray values are distributed based on the contents of the scan. The contrast of each individual scan is optimized, but gray values differ between scans containing low- or high-density materials. The presence of high-density objects in the scan shifts the histogram, leading to lower gray values throughout the image [28]. Previous studies tried to correct the inconsistency and calibrate gray values along a Hounsfield unit or a density scale [13, 28–30].

In this study, low- and medium-density values (air and a central FOV soft tissue) were used as reference calibration points for image normalization. The use of a reference object in the FOV containing at least two materials of known density could allow for a calibration similar to the use of reference phantoms in quantitative CT, rather than the “standard” automatic normalization function available in image analysis software that uses the lowest and highest gray values as calibration points. Although some differences in the rescaled values still exist because of differences in kilovoltage and filtration, they do provide a more meaningful result than the original gray values. Thus, rescaling might allow the comparison of densities from different patients, which would otherwise not be possible.

**Fig. 3:**
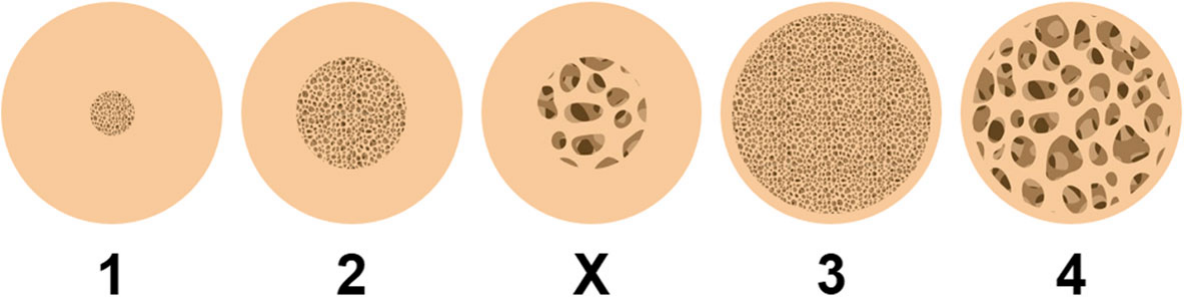
One possible missing category (depicted as “X”) would be a bone type with thick cortical layer and sparse trabecular bone, which would have intermediate characteristics and behavior between the types with thick (2) or thin (3) cortical bones

The average mean gray values were higher in the mandible than in the maxilla, like previously reported by González-Garcia and Monje [31], and premolar region and molar region, respectively, indicating anatomical differences for bone density. In comparison with insertion torque, the average mean gray values were different between the extreme categories of < 15 N cm and > 35 N cm, but not with the intermediate insertion torque. Although there was a consistent numerical decrease in absolute values, mean gray values were only statistically different for bone type 4 as classified by the subjective intra-surgical tactile evaluation. Bone types 2 and 3 were not discriminatory, which suggests inter-variability in these categories. One possible explanation is that the classification of bone quality was firstly determined by the tactile perception of the cortical bone thickness during the surgical perforation. A thick cortical layer would categorize the bone into types 1 or 2 according to the widely used classification by Lekholm and Zarb [5], even if the trabecular bone was not dense. One possible missing category would be a bone type with thick cortical layer and sparse trabecular bone, which could have intermediate characteristics and behavior between the types of thick and thin cortical bones (Fig. 3). This may be particularly important for short implants because the ROI, to determine the mean gray values and surgical perforation, had 6 mm in depth. Thus, the presence of a less-dense trabecular bone in some of the sites classified as bone type 2 may possibly have decreased the mean gray values. Fuster-Torres et al. [23] found significant differences between the maxilla and the mandible in mean bone density using Hounsfield unit (HU) in CBCT, with a mean torque insertion of 42.4 + − 4 N cm, with no differences for insertion torque (IT) between the posterior maxilla and mandibular implants, with very low bone density vs IT correlation coefficients. This could be related to the gray values (GV) inconsistency without GV calibration and using HU in CBCT. Comparisons between the present study and Fuster-Torres et al.’s study are not possible due to several method differences, including the use of HU in CBCT and different implant geometry, which alone is not adequate.

The present study also used the same preoperative CT images for visual evaluation of bone quality at the exact implant site, using the axial, coronal, and sagittal sections in a standardized procedure. There was a fairly moderate correlation between CT visual assessment and mean gray values, but no statistical difference was found in average mean gray values between the visual classification of bone types 3 and 4. One likely explanation is that the visual assessment is highly subjective to analyze bone structure and density in contrast to the objective quantification of minor changes in gray values invisible to the naked eye of the most experienced imaging specialist. The preoperative identification of bone type 4 implies greater surgical care and a certain risk for the prosthetic treatment. In these cases, possible clinical procedures include sub-drilling and bicortical anchoring, submerged, two-stage protocol, avoiding immediate or early loading, and using implants with surface treatment.

One limitation of the present study is the restriction to a single implant system and measurements and the relative small sample size, which decreased the power of analysis within the subgroups of bone types, although statistically significant results were found. Nevertheless, this study introduces a standardized method to assess bone quality in the exact implant site, using pre-surgical CT images in the axial, coronal, and sagittal sections. Further research is warranted to test the assessment methods in larger and diverse samples and to develop other three-dimensional analytical protocols in preoperative CT images for a possible “virtual biopsy” of the implant site.

## Conclusions

In summary, within the conditions and limitations of this study, the results suggest that bone quality has a significant effect on the primary stability of short implants as measured by insertion torque. Insertion torque had significant correlation with all assessment methods of bone quality. For preoperative CT evaluation of bone quality, mean gray values (optical density) had stronger association with insertion torque than subjective visual assessment. Therefore, preoperative quantification of bone quality with good correlation with surgery outcome measures could save clinical time and improve implant treatment planning.

## Abbreviations

CBCT: Cone beam computed tomography
CT: Computed tomographic
DICOM: Digital Imaging and Communications in Medicine
FOV: Field of view
MGVs: Mean gray values
ROI: Region of interest
